# Altered Spontaneous Brain Activity in Poststroke Aphasia: A Resting-State fMRI Study

**DOI:** 10.3390/brainsci13020300

**Published:** 2023-02-10

**Authors:** Haozheng Li, Hui Zhang, Shuai Xu, Mengxing Wang, Jilei Zhang, Jianren Liu, Xiaoxia Du, Ruiping Hu

**Affiliations:** 1 Department of Rehabilitation Medicine, Huashan Hospital, Fudan University, Shanghai 200040, China; 2 Shanghai Key Laboratory of Magnetic Resonance and Department of Physics, School of Physics and Materials Science, East China Normal University, Shanghai 200062, China; 3 Department of Neurology, Shanghai Ninth People’s Hospital, Shanghai Jiao Tong University School of Medicine, Shanghai 200011, China; 4 Department of Psychology, Shanghai University of Sport, Shanghai 200438, China

**Keywords:** poststroke aphasia, functional magnetic resonance imaging, regional homogeneity, low-frequency fluctuation, brain function

## Abstract

Purpose: Brain areas frequently implicated in language recovery after stroke comprise perilesional sites in the left hemisphere and homotopic regions in the right hemisphere. However, the neuronal mechanisms underlying language restoration are still largely unclear. Methods and materials: In the present study, we investigated the brain function in 15 patients with poststroke aphasia and 30 matched control subjects by combining the regional homogeneity (ReHo) and amplitudes of low-frequency fluctuation (ALFF) analysis methods based on resting-state fMRI. Results: Compared to the control subjects, the patients with aphasia exhibited increased ReHo and ALFF values in the ipsilateral perilesional areas and increased ReHo in the contralesional right middle frontal gyrus. Conclusions: The increased spontaneous brain activity in patients with poststroke aphasia during the recovery period, specifically in the ipsilateral perilesional regions and the homologous language regions of the right hemisphere, has potential implications for the treatment of patients with aphasia.

## 1. Introduction

Aphasia is a language disorder that is usually caused by left hemisphere stroke and is characterized by impairments in language comprehension, verbal expression, reading comprehension, and written expression. Although a significant proportion of patients with aphasia demonstrate some degree of recovery, a significant proportion of individuals continue to experience persistent language deficits. The lesion location and size and the aphasia type and severity are the most important factors that determine recovery [1]. However, how the bilateral hemispheres change or adapt is poorly understood. The selection of excitatory or inhibitory noninvasive brain stimulation, such as repetitive transcranial magnetic stimulation (rTMS) or transcranial direct current stimulation (tDCS), depends on the answer to this question.

Most studies have found that the recovery of the language function in aphasia is a very complex process [2]. Furthermore, studies have focused on the role of the left hemisphere; however, for the right hemisphere, whether the right hemisphere recruitment is compensatory [3,4] or maladaptive [5,6] in language recovery is still unclear. In a previous study, Fridriksson et al. suggested that increased activation in the preserved left hemisphere areas was associated with better naming performance in aphasia [7]. Meinzer et al. also highlighted the important role of left-hemispheric perilesional brain regions in language recovery in aphasia [8]. Previous studies have also investigated the right hemisphere’s structure and function changes in chronic left hemisphere stroke [3,9,10,11]. Changes in the right hemisphere structure and function are not a mere consequence of the damage to the left-sided homologous areas but are due to functional recruitment [11].

Structural MRI and functional MRI (fMRI) can be used to investigate language architecture and language-related brain activity to explore the neurobiological mechanism underlying poststroke aphasia [12,13,14,15]. A resting-state functional MRI has been used to examine functional connectivity within language networks [16,17,18]. Although the functional connections can identify brain networks, including various brain regions from the whole brain, they cannot reveal the regional brain’s spontaneous neuronal activity. Amplitude low-frequency fluctuation (ALFF) and regional homogeneity (ReHo) analysis methods are data-driven methods often used to investigate spontaneous neuronal activity in specific brain regions in resting-state fMRI studies [19]. One study reported that, compared to healthy control subjects, poststroke aphasia patients exhibited a disturbed ReHo in the right lingual gyrus, left calcarine sulcus, left cuneus, and left superior frontal gyrus in the resting state [20]. Meanwhile, ALFF analysis has been used to investigate the brain mechanisms responsible for treatment-induced recovery [21]. Yang et al. found that patients with aphasia showed significantly increased ALFF values, mainly in the contralateral mesial temporal and lateral temporal cortices [22]. To date, information regarding regional brain spontaneous neuronal activity under the resting state within the left perilesional and right hemisphere homologous areas in poststroke aphasia has remained largely unavailable.

In this study, we analyzed resting-state fMRI data to evaluate the dysfunction of the spontaneous brain activity in patients with poststroke aphasia by applying the ReHo and ALFF analysis methods. The ReHo method measures the regional similarities or synchronizations of temporal changes in the blood oxygen level-dependent (BOLD) activity by calculating Kendall’s coefficient of concordance (KCC) [23]. ALFF analysis is effective for detecting fluctuations in spontaneous low-frequency oscillations, and ALFF changes are thought to be associated with regional spontaneous neuronal activity. In the present study, we investigated whole-brain (especially the left perilesional and right hemisphere homologous areas) spontaneous activity in patients with poststroke aphasia by ReHo and ALFF analysis using resting-state fMRI. Structural and functional (under task) changes have been observed in the left perilesional and right hemisphere homologous areas of poststroke aphasia patients in previous studies [3,7,9,10,12]. Thus, we hypothesized that spontaneous brain activity in the left perilesional and right hemisphere homologous areas would show adaptive changes during the subacute and chronic phases of poststroke aphasia. The spontaneous brain activity is measured from resting-state fMRI without task; thus, the collection of data is relatively simple compared to the task fMRI, including patients with global aphasia or in the acute phase. Currently, it is very promising that resting-state fMRI can be used to localize language networks to help neurosurgeons perform surgery to avoid aphasia [24,25]. In the field of poststroke aphasia research, results from resting-state fMRI can be used to help localize stimulation targets for noninvasive brain stimulation [26] and to monitor the effectiveness of treatment [21,27]. Our study has the potential to help physicians to select the appropriate treatment for those with aphasia and to monitor the effects of treatment.

## 2. Materials and Methods

### 2.1. Subjects

Fifteen patients with aphasia (12 males, 3 females, aged 50.60 ± 7.86 years old) were recruited upon admission to Shanghai Huashan Hospital. For all patients, this was the first stroke, and the lesion was located in the left hemisphere. Aphasia was diagnosed by the Western Aphasia Battery (Simplified Chinese version) (C-WAB) [28]. Healthy controls with age and sex 1:2 matched with patients (24 males, 6 females, aged 50.10 ± 7.95 years old) were recruited. All subjects were right-handed. Participants were excluded due to any neurological or psychiatric disease or a history of head injury or substance abuse (Table 1).

This study was approved by the Ethics Committee of Huashan Hospital. All participants or their legal family members provided written informed consent using forms approved by the committee.

### 2.2. MRI Acquisition

Structural MRI and fMRI data were collected by a 3T Trio Tim Siemens MR scanner (Siemens, Erlangen, Germany) equipped with a 12-channel head coil. Custom-fit foam pads limited the head motion in the coil. The whole-brain anatomical volume was determined using a T1-weighted, high-resolution, three-dimensional, magnetization-prepared gradient-echo pulse sequence with rapid acquisition. The T1-weighted image parameters were as follows: sagittal orientation, field of view 256 × 256 mm^2^, matrix size 256 × 256, 192 slices with thickness 1 mm, repetition time 2530 ms, echo time 2.34 ms, inversion time 1100 ms, and flip angle 7°. The resting-state fMRI images were acquired using a T2*-weighted gradient-echo echo-planar imaging pulse sequence with the following parameters: repetition time 2000 ms, echo time 30 ms, flip angle 90°, number of slices 33, transverse orientation, field of view 220 × 220 mm^2^, matrix size 64 × 64, slice thickness 3.5 mm, 25% distance factor, and a total of 210 volumes. The duration of the resting-state sequence was 7 min. During the fMRI scan, the subjects were instructed to remain still, close their eyes, and relax.

### 2.3. Lesion Mapping

**Figure 1:**
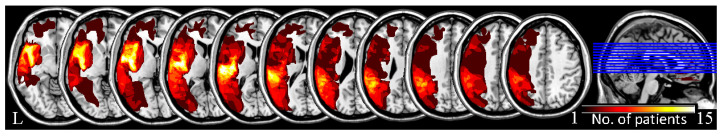
The lesion overlap images for all patients with aphasia.

The investigator manually traced the outline of the lesion on individual 3D T1 images using MRIcron, thereby creating a lesion mask for each patient. After the spatial normalization process, all the individual lesion masks were combined to construct a group lesion mask for each patient (Figure 1) [22]. A rigorous approach was adopted in the group lesion mask, whereby voxels that were identified as lesioned in only one participant were excluded from the entire analysis in all participants.

### 2.4. Resting-State fMRI DATA Preprocessing

fMRI data preprocessing was carried out using the Data Processing Assistant for Resting-State fMRI (DPARSF) (http://www.restfmri.net, accessed on 22 May 2022) based on statistical parametric mapping software (SPM12; http://www.fil.ion.ucl.ac.uk/spm/software/spm12, accessed on 22 May 2022) and MATLAB (The Math Works, Natick, MA, USA) software. For each participant, the first 10 volumes were removed to allow the participants to adapt to the scanner noise and magnetization equilibration. Then, the acquisition time of the remaining volumes was corrected to a value similar to that of the middle slice of each volume, and all the volumes were realigned to the first volume using the six-parameter (rigid body) spatial transformation to correct for head motion of the participants during the fMRI scan. After these corrections, the high-resolution T1-weighted image was co-registered to the mean functional image. The fMRI images were spatially normalized to the standard Montreal Neurological Institute (MNI) template by Diffeomorphic Anatomical Registration Through Exponentiated Lie Algebra (DARTEL) [29] based on the high-resolution T1-weighted images and resampled to 3 × 3 × 3 mm^3^. Finally, six head motion parameters and the signal from both the white matter and the cerebrospinal fluid were regressed out using a general linear model, and the linear trends were removed from the fMRI data as well.

The head motion parameters of all the participants were calculated, including 3 translational and 3 rotational motion parameters. Participants were excluded if their head movement exceeded a 2 mm translation or a 2° angular rotation in any axis; none of the participants exhibited excessive movement. In addition, no significant differences were found between the head movement data of the two groups of subjects after a two-sample t-test.

### 2.5. ReHo and ALFF Analysis

DPARSF (http://www.restfmri.net, accessed on 22 May 2022) was used to conduct the ReHo and ALFF analyses. To measure the ReHo, band-pass filtering (0.01–0.08 Hz) on the preprocessed data was applied to reduce the influence of high-frequency respiratory and cardiac noise as well as low-frequency drift. An individual ReHo map was quantified by the KCC between a voxel and its 26 neighbors [23]. Then, the ReHo value of each voxel was converted into a z-score by subtracting the mean ReHo value and dividing this value by the standard deviation of the whole-brain ReHo map. Finally, smoothing was conducted with a 6 mm full width at half maximum (FWHM) Gaussian kernel for standardized ReHo maps.

To calculate the ALFF, we first performed spatial smoothing on the preprocessed data with a 6 mm FWHM Gaussian kernel. Then, for each voxel, the time series was transformed to the frequency domain via fast Fourier transforms, and the square root of the power spectrum was calculated and then averaged across 0.01–0.08 Hz; the final value was defined as the ALFF. Finally, all the ALFF maps were converted into a standardized z-score by subtracting the mean ALFF value and dividing the new result by the standard deviation of the whole-brain ALFF map.

### 2.6. Correlation Analysis

A multiple regression was used to calculate the relationship between the spontaneous brain activity and the clinical data by SPSS software version 26.0 (IBM Corporation, Armonk, NY, USA). The clinical data included spontaneous speech, auditory comprehension, repetition, naming, and aphasia quotient (AQ) scores extracted as the dependent variables Y, and the lesion size, time post-stroke (months), and the individual mean ReHo and ALFF z-scores, extracted as the predictor variables (X).

## 3. Results

### 3.1. Clinical Data for Patients with Aphasia

Table 2 provides the details regarding the C-WAB score, which includes the score of the different dimensions of language and aphasia quotient. The type of aphasia was assessed according to the spontaneous speech, auditory comprehension, and repetition scores, and the AQ reflects a global measure of the severity of the aphasia.

### 3.2. ReHo and ALFF

**Figure 2:**
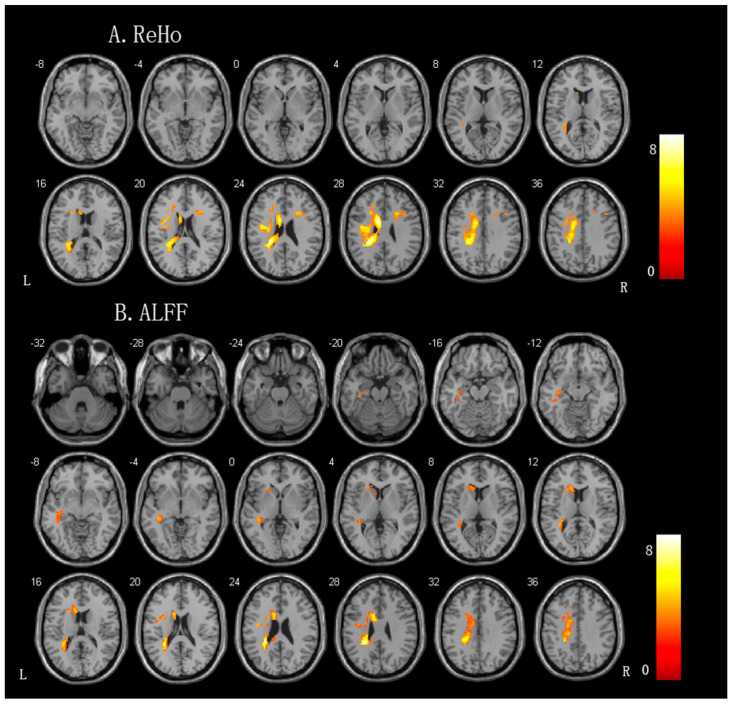
Brain regions showing significant differences in the spontaneous brain activity between the patients with aphasia and controls. (**A**) ReHo (regional homogeneity) value. (**B**) ALFF (amplitudes of low-frequency fluctuation) value.

Compared to the controls, the patients with aphasia exhibited significantly increased ReHo values in the left perilesional areas, including the left cingulate gyrus, the left frontal lobe, the left sublobar region, the left precentral gyrus, and the right middle frontal gyrus. The ALFF changes were similar to the ReHo changes; specifically, patients with aphasia exhibited significantly increased ALFF values in the left frontal lobe, left sublobar region, left corpus callosum, left cingulate gyrus, left temporal gyrus, and right cerebellum posterior lobe (for more details, please see Figure 2 and Table 3).

### 3.3. Correlations between the ReHo and the Clinical Scores

No significant results were observed between the clinical data and the spontaneous brain activity (*p* > 0.05).

## 4. Discussion

Our results showed that the patients with aphasia exhibited increased ReHo and ALFF values in the ipsilateral perilesional areas and increased ReHo in the contralesional right middle frontal gyrus. The recovery of language function after a stroke has been associated with the reorganization of language networks; this recovery can take the form of the recruitment of new regions ipsilateral to the lesion or a shift of language processing to the right nondominant hemisphere [30]. In a previous resting-state MEG study, Shah-Basak et al. found that stronger alpha and beta connectivity was associated with better language performance in patients with aphasia [31]. However, this abnormal signal does not necessarily come from neuroplasticity; the abnormal signals could also reflect pathological activity, through such mechanisms as selective neuronal loss [32], restricted blood flow [33], or disconnection [34]. Many EEG/MEG studies have shown elevated low-frequency activity in stroke patients [35,36]. These abnormalities could be pathological as well as compensatory.

In our study, the alteration of spontaneous brain activity was mostly located in the perilesional areas of the left hemisphere in patients with aphasia. Mohr et al. found that after two weeks of intensive language action therapy, the enhancement of brain activation was most pronounced in the peripheral over the perilesional areas in the left hemisphere [37]. Fridriksson et al. also proposed that left hemisphere plasticity plays an important role in aphasia recovery, and their findings suggested that improved naming was associated with the modulation of the left frontal lobe by behavioral anomia treatment in aphasia [38]. Previous studies have posited that the reactivation of preserved language regions within the left hemisphere may lead to optimal clinical outcomes [39,40,41,42]. In some TMS studies, it was found that stimulating the task fMRI hotspots located in the peripheral regions of the left hemisphere could lead to improvements in language for individuals with aphasia [43,44,45].

The patients with aphasia exhibited increased spontaneous brain activity in the left cingulate gyrus, left frontal lobe, left sublobar region, left precentral gyrus, left corpus callosum, left temporal gyrus, and right cerebellum posterior lobe. The default mode network has been shown to be abnormal after stroke [46], and a study by Sandberg et al. reported that people with aphasia showed a pattern of hypoconnectivity in the default mode network during the chronic phase compared to healthy controls [47]. This was similar to the results of another study that included both people with subacute- and chronic-phase aphasia [48], which may also explain the reason we did not find increased spontaneous activity in the default mode network. These areas are mostly in the language network [16]. Language processing requires the recruitment of a complex network including the temporal, frontal, and parietal brain regions; then, language recovery depends on the reorganization processes within these networks. The increased spontaneous brain activity in the left hemisphere remote and perilesional areas may suggest a gradual reintegration and contribution to language recovery. It has been demonstrated through various studies that poststroke neuroplasticity can occur in perilesional regions as well as in areas distant from the initial lesion [38,49]. Our results may reflect improvements in cortical function due to spontaneous rehabilitation over time after stroke.

We also investigated the increased ReHo in the contralesional right middle frontal gyrus. The right middle frontal gyrus is the right hemisphere homologous region of the left hemisphere language areas, which may play an integral role during recovery from aphasia [16]. Previous studies have documented elevated activity within the right hemisphere in comparison to healthy controls during language-related tasks [50,51,52], which suggested the compensatory role of the right hemisphere. Uruma et al. reported that the regional cerebral blood flow decreased in the left Brodmann area 22 and significantly influenced the regional cerebral blood flow increase in the right language-relevant regions [53]. It is noteworthy that all people with aphasia in our study were native speakers of Mandarin, and the frontal middle gyrus is very important for Chinese [54]; the increase in white matter volume in the frontal middle gyrus was significantly associated with Chinese reading ability but not with English reading ability [55]. Reading ability is important for Chinese characters, which may explain the increased ReHo in the right frontal middle gyrus of Chinese people with aphasia. This may reveal that despite the increased activation of the right hemisphere during language reorganization in aphasic speakers, there are differences in location across languages [56].

Our study showed increased spontaneous brain activity in both hemispheres in patients with poststroke aphasia, which suggested that both hemispheres contributed to functional recovery. The patients with aphasia showed increased spontaneous brain activity, mostly in the left perilesional areas and a few in the right hemisphere homologues. The investigation of the dynamics of the reorganization of the language system in patients with aphasia from the acute to the chronic stage by repeated task fMRI examinations by Saur et al. found that in the acute phase, the patients showed little early activation of the remaining left language areas; whereas, in the subacute phase, the patients showed a large increase in the activation of the bilateral language network with the recruitment of the homologue language zone. In the chronic phase, a normalization of activation was observed with a re-shift in the peak activation to the left hemispheric language areas [57]. Previous studies have revealed that the perilesional areas in the spared left hemisphere, as well as the contralesional right hemisphere regions, all made functional contributions to language recovery changes over time [2,58]. A prior study discovered that there was a positive correlation between the lesion size and the increased activity in the right hemisphere during language tasks. This finding suggests that the extent to which reorganization contributes to the recovery from aphasia is contingent upon the size and location of the lesion [59]. Most previous studies have focused on task-state functional MRI, which is limited by the necessity of having a language task that all aphasic individuals can perform. Language tasks generate more activity in both hemispheres in both healthy controls and patients with aphasia [60]. Our study used resting-state MRI, which means that aphasic individuals can reflect spontaneous brain activity without the effort of completing the language task. Our study provides complementary evidence of enhanced bilateral spontaneous brain activity in patients with aphasia during the recovery period.

In a language model, mild aphasia is closely related to the retention of the primary language area. Patients in this category recover best and involve weak right brain activation. In contrast, moderate aphasia is associated with more extensive but incomplete lesions in the major language areas, and patients have strong right-brain activation in the subacute phase and a shift to the left hemisphere in the chronic phase. In severe aphasia, where the major language areas are almost completely destroyed, patients have substantial right hemisphere activation in the subacute and chronic phases, and they have very limited recovery, usually maintaining only a lower level of language [61]. In patients with stroke, a “bimodal balance recovery” model was proposed to define the role of the bilateral hemispheres [62], which is considered to depend mainly on the severity of the patient’s symptoms, and Lin et al. [63] confirmed this hypothesis in patients with poststroke motor dysfunction and provided a clinical score threshold for performing stratification. Whether such a model also exists in patients with aphasia may depend on future studies.

Whereas our investigation revealed elevated levels of spontaneous brain activity in individuals with poststroke aphasia, it should be acknowledged that there were several limitations in the present study. First, our study mixed patients with aphasia in the subacute and chronic phases, as bilateral recruitment may depend on the time after stroke. Moreover, our study did not find a statistically significant association between the spontaneous brain activity of people with aphasia and their language performance, which may be due to the complexity of the relationship between the partial brain activity and the degree of language retention. Further, the aphasic group’s sample size was relativity modest, which limited the statistical power and the strong interpretation of the nonsignificant association between the brain activity and the language scores. We will use stricter inclusion criteria in future studies to reduce the variation between patients and increase our sample size to eliminate these concerns. In addition, a conservative approach was adopted for lesion management. Voxels that were identified as lesioned in only one participant were excluded from the entire analysis, resulting in an inability to determine whether the changes in the spontaneous brain activity were confined to specific regions of the left hemisphere. The limited data that remained after the exclusion only encompassed restricted cortical or subcortical/white matter areas of the left hemisphere, as most of the left hemisphere cortex was lesioned in at least one subject. Finally, this was a cross-sectional study; a longitudinal study would be more useful to uncover the functional dynamic changes during stroke recovery.

## 5. Conclusions

This study presented evidence of heightened levels of spontaneous brain activity in patients with poststroke aphasia during the recovery period, specifically in the ipsilateral perilesional regions and the homologous language regions of the right hemisphere. These results have the potential to inform treatment strategies for individuals with aphasia.

## Figures and Tables

**Table 1:** Demographics of the patients with aphasia and healthy controls.

	Aphasia (*n* = 15)	Healthy Control (*n* = 30)
Handedness (left/right)	0/15	0/30
Male/Female	12/3	24/6
Age (years)/mean ± SD	50.60 ± 7.86	50.10 ± 7.95

**Table 2:** The Western Aphasia Battery test scores for patients with aphasia.

ID	Sex/Age (Years)	Time Post Stroke (Months)	Spontaneous Speech	Auditory Comprehension	Repetition	Naming	AQ	Aphasia Type	Lesion Size (Voxel)
1	M/38	10	14/20	6.65/10	10/10	5.7/10	72.7/100	T.S.	142,981
2	F/39	4	17/20	10/10	6.6/10	8/10	83.2/100	Conduction	25,678
3	M/41	5	16/20	9.65/10	8.1/10	8.3/10	84.1/100	Broca’s	28,098
4	M/42	3	6/20	1.05/10	1.7/10	0.1/10	17.7/100	Wernicke’s	93,266
5	M/47	14	5/20	5.75/10	3.8/10	4.2/10	37.5/100	Broca’s	38,237
6	M/48	2.4	7/20	6/10	4.2/10	6.5/10	47.4/100	Broca’s	35,934
7	F/51	28	0/20	5.6/10	0/10	0/10	11.2/100	Broca’s	59,442
8	M/52	1.2	2/20	7.1/10	0/10	0/10	19.2/100	Broca’s	57,237
9	M/54	21	16/20	7.3/10	4.5/10	6.1/10	67.8/100	Conduction	21,080
10	F/54	3	2/20	6.55/10	0.6/10	0/10	19.3/100	Broca’s	18,821
11	M/55	6	10/20	4.2/10	7.8/10	1.8/10	47.6/100	Wernicke’s	21,419
12	M/57	6	6/20	4.1/10	3.6/10	0.6/10	28.6/100	Broca’s	57,237
13	M/57	13.5	7/20	8.2/10	8.6/10	1.2/10	25/100	T.M.	33,687
14	M/62	4.6	12/20	7.1/10	5.2/10	2.9/10	52.7/100	Anomic	89,642
15	M/62	2	0/20	0.9/10	0/10	0/10	1.8/100	Global	85,145

Note: M, male; F, female, AQ, aphasia quotient; T.S., transcortical sensory; T.M., transcortical motor.

**Table 3:** The ReHo and ALFF values were increased in the patients with aphasia compared to those in the healthy controls.

Brain Regions	Number of Voxels	T Value	Peak Location			*p* Value
			X	Y	Z	
**ReHo increase in aphasia group**						
**Left cingulate gyrus**	**899**	**8.24**	**−9**	**0**	**27**	**0.001**
Left frontal lobe		7.94	−21	−33	27	
Left sublobar		4.77	−24	18	18	
Left precentral gyrus		4.07	−33	−3	27	
**Right frontal lobe**	**120**	**4.89**	**24**	**15**	**27**	**0.002**
Right middle frontal gyrus		4.43	24	21	45	
**ALFF increase in aphasia group**						
**Left frontal lobe**	**770**	**9.40**	**−24**	**−39**	**27**	**0.001**
Left sublobar		7.89	−30	−42	18	
Left corpus callosum		6.44	−9	9	27	
Left cingulate gyrus		5.64	−18	−24	36	
Left temporal lobe		4.45	−39	−30	−6	

Note: *p* < 0.001 uncorrected at voxel level, cluster size >30, and cluster level FWE (family wise error) corrected. X, Y, Z: Montreal Neurological Institute; the bolded words represent the largest size in a cluster.

## Data Availability

The data presented in this study are available on request from the corresponding author. The data are not publicly available due to privacy or ethics.
